# Association between plain water intake and risk of hypertension: longitudinal analyses from the China Health and Nutrition Survey

**DOI:** 10.3389/fpubh.2023.1280653

**Published:** 2024-01-09

**Authors:** Shizhen Li, Xun Xiao, Xiangyu Zhang

**Affiliations:** Department of Geriatrics, The Second Xiangya Hospital, Central South University, Changsha, Hunan, China

**Keywords:** plain water intake, hydration, hypertension, longitudinal cohort, China Health and Nutrition Survey

## Abstract

**Objective:**

This study aimed to investigate the prospective association between plain water intake and the risk of hypertension based on a longitudinal cohort study in China.

**Methods:**

Logistic regression analyses were performed to investigate the association between plain water intake and hypertension. Restricted cubic spline model was use to evaluate non-linear relationship between plain water intake and hypertension. Subgroup analyses and interaction tests were conducted based on age, gender, residence site, educational level and tea consumption.

**Results:**

A total of 3,823 participants (46.5% male) with a mean age of 46.8 years from the China Health and Nutrition Survey (CHNS) were assessed and divided into 4 groups based on plain water intake. There was a decreasing trend of hypertension risk as plain water intake increased. Logistic regression analyses indicated that participants consuming plain water ≥6 cups/day (1 cup ≈ 240 mL) had significantly lower risk of hypertension compared to those consuming ≤1 cup/day, even after adjustments for covariates. Restricted cubic spline curve revealed that participants consuming about 6–8 cups/day were at lower risk for developing hypertension. In subgroup analyses, the results were generally consistent with the main findings in participants who aged less than 60 years, who were male, who attained higher education and who were low tea consumers.

**Conclusion:**

Our findings suggested that there might be a favorable effect of plain water intake on preventing hypertension in a large cohort of Chinese adults from the general population. Drinking adequate amounts of plain water (about 6–8 cups/day) may reduce the risk of hypertension, particularly in the selected population. Further interventional studies are required to investigate the potential effect of increasing plain water intake on blood pressure regulation.

## 1 Introduction

Hypertension represents an increasing global disease burden affecting nearly one third population worldwide (1). It is an important risk factor for incidence and mortality of cardiovascular disease. Despite the increasing prevalence, the proportions of awareness, treatment and control of hypertension are low (2). The etiology of hypertension is complex and involves environmental and pathophysiological factors, as well as genetic conditions (3). Hypertensive patients usually require both lifestyle and pharmacologic intervention. Healthy lifestyle interventions, including healthy diet, normal body weight, exercise training, and non-smoking, are recommended to prevent or control early stage of hypertension and proved to provide health benefits for hypertensive patients (4, 5).

Adequate plain water intake is essential for normal functioning of the human body (6). Recent evidence has demonstrated that maintaining appropriate hydration status is an integral part of healthy lifestyle behaviors for the prevention and management of metabolic diseases (7, 8). Observational studies also suggest that appropriate hydration status could decrease the risk of age-related diseases (9–12). Furthermore, plain water substituting for sugar-sweetened beverages was associated with lower risk of cardiovascular mortality in diabetic patients (13). However, there is no population-based evidence about the association between plain water intake and risk of hypertension. It remains unknown whether high water intake has a favorable effect on blood pressure control. In the present study, we aimed to investigate the association between plain water intake and the risk of hypertension during a follow-up period of 9-year in China Health and Nutrition Survey (CHNS) among Chinese adults.

## 2 Materials and methods

### 2.1 Study design and population selection

The CHNS is a population-based longitudinal survey with a multistage, random cluster design across nine provinces (including Jiangsu, Hubei, Hunan, Guangxi, Guizhou, Heilongjiang, Liaoning, Shandong, and Henan) in China. Based on the Qinling Mountains–Huaihe River Line, China is divided into North China and South China geographic areas (14). The nine provinces selected in the original CHNS are distributed in these two regions, Heilongjiang, Liaoning, Shandong and Henan in northern China; Jiangsu, Hubei, Hunan, Guangxi and Guizhou in southern China. All the analyses in this study were based on the data in the wave of 2006 and 2015. Further information on survey procedures and the rationale of the CHNS is in the cohort profile and available at the website.^1^ The survey received ethical approval by the Institutional Review Board of the University of North Carolina at Chapel Hill, the National Institute for Nutrition and Food safety at China Center for Disease Control and Prevention, and the Human and Clinical Research Ethics Committee of the China-Japan Friendship Hospital. All participants signed the written informed consents. The detailed information of the CHNS has been published elsewhere (15).

**Figure 1:**
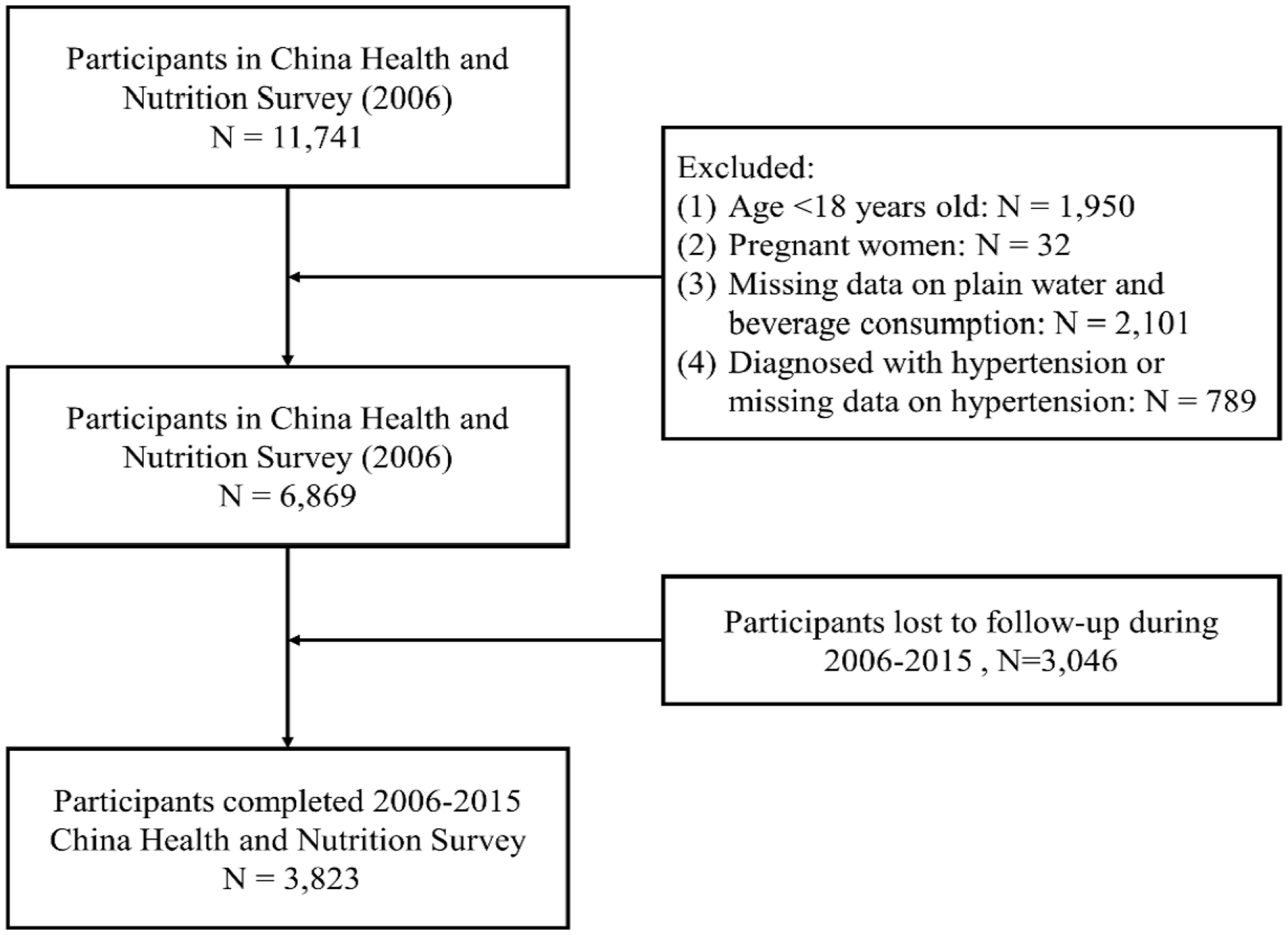
The flow diagram of participant selection.

A total of 11,741 participants in the wave of 2006 from CHNS were enrolled. The exclusion criteria of the present study included age less than 18 years old (*n* = 1,950), pregnancy (*n* = 32), missing data on plain water and beverage consumption (*n* = 2,101), and diagnosed with hypertension or missing data on hypertension (*n* = 789). 3,046 participants lost follow up in the wave of 2015, and 3,823 participants were finally included in the study. The procedure of population selection was depicted in Figure 1.

### 2.2 Assessment of plain water intake

A frequency questionnaire and the China food composition tables were used to collect daily water intake in the wave of 2006. The questions, “How often did you drink water during the past 30 days?” and “How many cups (1 cup ≈ 240 mL) did you drink per day?” were used for calculating the consumption of plain water.

### 2.3 Ascertainment of hypertension

Hypertension was identified by the questionnaire-based interview in two surveys in the wave of 2006 and 2015. The question involved in these questionnaires was to collect individual information about the history of hypertension: Has a doctor ever told you that you suffer from high blood pressure? Answering “yes” to the question was defined as having self-reported diagnosis of hypertension and identified with new-onset hypertension in the following survey in 2015.

### 2.4 Covariates

Demographic data and lifestyle information were obtained by the CHNS questionnaire and physical examination including age, gender, height, weight, beverage consumption, diabetes mellitus, myocardial infarction and stroke. Body mass index (BMI) was calculated as weight (kg)/ height^2^ (m^2^), and categorized as ≥24 or < 24 kg/m^2^ group to evaluate potential effect of overweight/obesity. Smoking status was categorized as past or current smoker, and never. Urbanization was categorized as urban and rural residence. Education level was categorized as junior high school or below, and senior high school or above. Energy intake was calculated from 3-day dietary-recall Chinese food composition tables, and categorized as ≥2,147 or < 2,147 kcal/day group by the median value across participants. Beverage consumption contained alcohol, tea, coffee, soft drinks and sugared fruit drinks. Diabetes mellitus, myocardial infarction and stroke was derived from the questions “Has a doctor ever told you that you suffer from diabetes mellitus, myocardial infarction, or stroke?”

### 2.5 Analysis

Continuous variables are presented as mean (standard deviation) or median (interquartile range) according to the distribution; categorical variables are presented as frequency (percentage). The trend differences across groups were tested by regression analysis for continuous variables and Cochran-Armitage tests for categorical variables. Multivariable logistic regression models were performed to explore whether plain water intake influenced onset risk of hypertension independently. Model 1 was only adjusted by age (≥ 60 or < 60 years), gender (male or female) and BMI (≥ 24 or < 24 kg/m^2^). Model 2 was adjusted for factors from Model 1 plus residence site (urban or rural), geographical region (North China and South China), education level (junior high school or below, or senior high school or above), energy intake (≥ 2,147 or < 2,147 kcal/day), smoking status (former or current smoker, or non-smoker), alcohol consumption (yes or no), tea consumption (more than 4–5 times/week or less than 2–3 times/week), coffee consumption (yes or no), and soft drinks or sugared fruit drinks consumption (yes or no). Restricted cubic spline analysis was used to evaluate the dose–response relationship between plain water intake and hypertension. Subgroup analyses based on age, gender, residence site, educational levels and tea consumption were used to evaluate the relationship between plain water intake and hypertension in these subgroups and the potential interaction between plain water intake and these stratified variables. All analyses were performed with R version 4.1.3 (The R Foundation for Statistical Computing, Vienna, Austria). A two-tailed *p* value <0.05 was determined to be statistically significant.

## 3 Results

A total of 3,823 Chinese adults with a mean age of 46.8 years and 1777 (46.5%) men were finally included in this study. Participants were divided into four groups: ≤ 1 cup/day, 2–3 cups/day, 4–5 cups/day and ≥ 6 cups/day according to self-reported plain water intake. Demographic and clinical characteristics of all participants across four groups were displayed in Table 1. There was an increasing trend in the proportion of males and urban residence as plain water intake increased. Participants consuming more plain water tended to be younger and educated. The incidence of hypertension was decreased across water intake groups, but not observed in diabetes mellitus, myocardial infarction and stroke. Since plain water and tea intake accounted for the most part of daily fluid intake, participants consuming less plain water were likely to drink more tea. As for other beverages, it was relatively evenly distributed in different water intake groups.

**Table 1:** Baseline characteristics of participants according to plain water intake.

Variables	Total	≤ 1 cup/day	2–3 cups/day	4–5 cups/day	≥ 6 cups/day	*p* for trend
Participants, *n* (%)	3,823 (100.0)	612 (16.0)	2009 (52.6)	888 (23.2)	314 (8.2)	
Age, years	46.8 (12.8)	48.6 (13.0)	46.9 (12.9)	45.7 (12.5)	45.9 (12.4)	<0.001
Men, *n* (%)	1777 (46.5)	259 (42.3)	935 (46.5)	417 (47.0)	166 (52.9)	0.006
BMI, kg/m^2^	23.1 (3.2)	22.9 (3.2)	23.1 (3.3)	23.1 (3.1)	23.2 (3.3)	0.222
Residence site, *n* (%)						<0.001
Urban	1,044 (27.3)	109 (19.8)	520 (25.8)	301 (33.5)	114 (36.1)	
Rural	2,779 (72.7)	503 (80.2)	1,489 (74.2)	587 (66.5)	200 (63.9)	
Geographical region						0.895
North China	1,659 (43.4)	266 (43.5)	859 (42.8)	413 (46.5)	121 (38.5)	
South China	2,164 (56.6)	346 (56.5)	1,150 (57.2)	475 (53.5)	193 (61.5)	
Educational level, *n* (%)						<0.001
Junior high school or below	1,650 (43.2)	295 (48.2)	909 (45.2)	325 (36.6)	121 (38.5)	
Senior high school or above	2,173 (56.8)	317 (51.8)	1,100 (54.8)	563 (63.4)	193 (61.5)	
Energy intake, kcal/day	2,147 (1731, 2,603)	2,100 (1713, 2,577)	2,147 (1718, 2,639)	2,150 (1746, 2,538)	2,205 (1821, 2,592)	0.888
Former or current smoker, *n* (%)	1,182 (30.9)	186 (30.4)	623 (31.0)	266 (30.0)	107 (34.1)	0.534
Alcohol drinking, *n* (%)	1,263 (33.0)	182 (29.7)	684 (34.0)	286 (32.2)	111 (35.4)	0.249
Diabetes mellitus, *n* (%)	26 (0.7)	5 (0.8)	10 (0.5)	9 (1.0)	2 (0.6)	0.655
Myocardial infarction, *n* (%)	9 (0.2)	0 (0.0)	5 (0.2)	2 (0.2)	2 (0.6)	0.113
Stroke, *n* (%)	7 (0.2)	2 (0.3)	5 (0.2)	0 (0.0)	0 (0.0)	0.090
Hypertension, *n* (%)	530 (13.9)	101 (16.5)	278 (13.8)	118 (13.3)	33 (10.5)	0.015
Tea consumption, *n* (%)						<0.001
More than 4–5 times/week	757 (19.8)	187 (30.6)	426 (21.2)	112 (12.6)	32 (10.2)	
Less than 2–3 times/week	3,066 (80.2)	425 (69.4)	1,583 (78.8)	776 (87.4)	282 (89.8)	
Coffee consumption, *n* (%)						0.984
Yes	47 (1.2)	9 (1.5)	23 (1.1)	10 (1.1)	5 (1.6)	
No	3,776 (98.8)	603 (98.5)	1986 (98.9)	878 (98.9)	309 (98.4)	
Soft drinks or sugared fruit drinks consumption, *n* (%)						0.095
Yes	878 (23.0)	112 (18.3)	484 (24.1)	209 (23.5)	73 (23.2)	
No	2,945 (77.0)	500 (81.7)	1,525 (75.9)	679 (76.5)	241 (76.8)	

BMI, body mass index. Continuous variables are presented as mean (standard deviation) or median (interquartile range) according to the distribution; categorical variables are presented as frequency (percentage). The trend differences across groups were tested by regression analysis for continuous variates and Cochran-Armitage tests for categorical variates.

**Figure 2:**
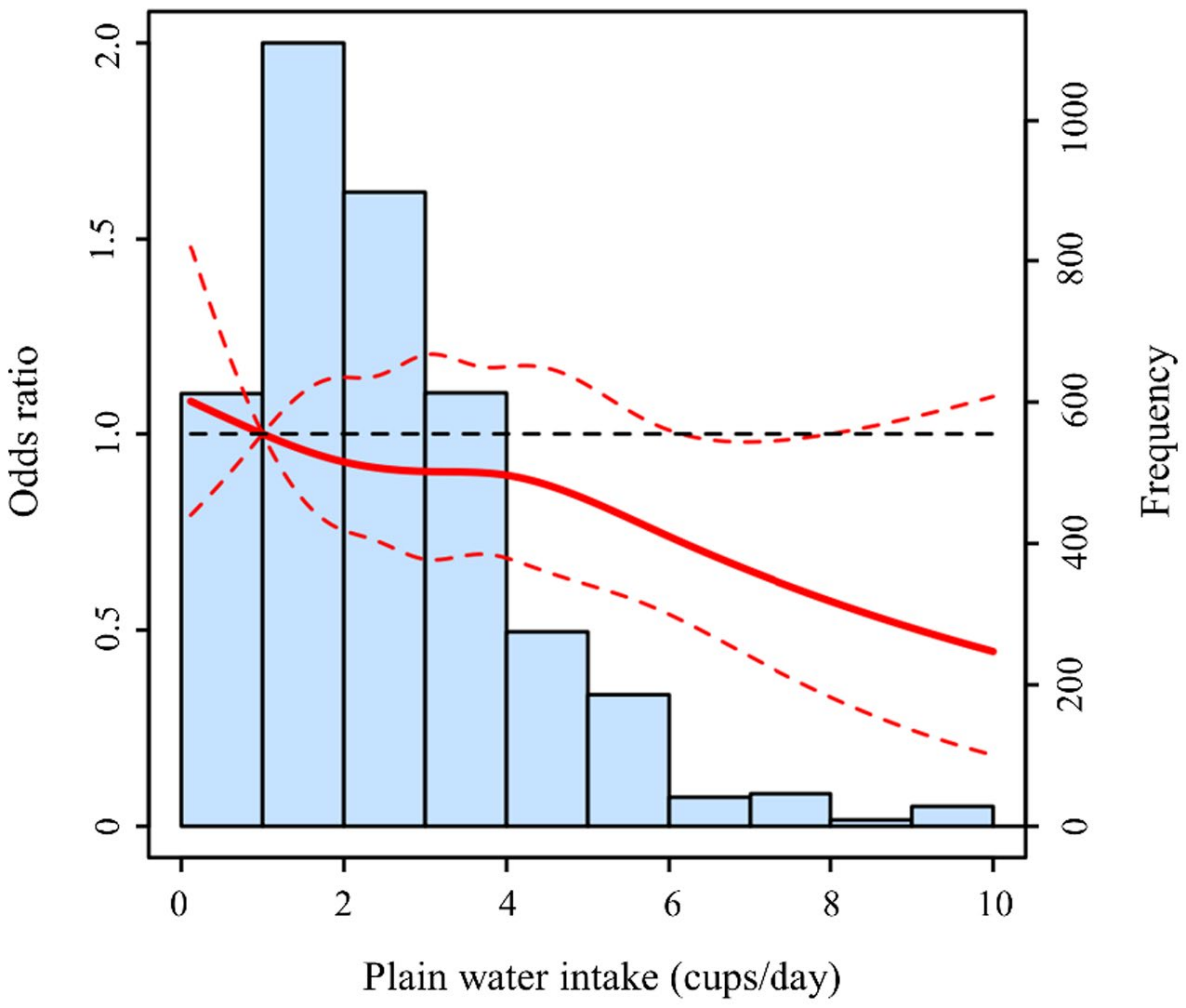
Non-linear association between plain water intake and hypertension risk in restricted cubic spline model. The red solid line represented OR, and the red dashed lines represented 95% CI. The model was adjusted by age, gender, BMI, residence, educational level, energy intake, smoking, alcohol consumption, and other beverages consumption.

To further explore the relationship between plain water intake and risk of hypertension, multivariate logistic regression analyses were conducted and summarized in Table 2. Taking the ≤ 1 cup/day group as reference, the unadjusted ORs (95% CIs) for 2–3 cups/day, 4–5 cups/day and ≥ 6 cups/day groups were 0.81 (0.64, 1.05), 0.78 (0.58, 1.04), and 0.59 (0.39, 0.89), respectively. There was a decreasing trend for hypertension risk across these groups. Moreover, the results remained similar after adjustments for corresponding covariates in both adjusted model 1 and model 2. As shown in Figure 2, there was no significant non-linear association (*P*-nonlinear >0.05) between plain water intake and hypertension displayed by restricted cubic spline curve. However, participants consuming about 6–8 cups/day were at lower risk for developing hypertension.

**Table 2:** Association between consumption of plain water intake and the risk of hypertension in the 9-year period cohort: CHNS 2006–2015.

	Crude model		Adjusted model 1		Adjusted mode 2	
Plain water intake	OR (95% CI)	*p* value	OR (95% CI)	*p* value	OR (95% CI)	*p* value
≤ 1 cup/day	Reference		Reference		Reference	
2–3 cups/day	0.81 (0.64, 1.05)	0.101	0.82 (0.64, 1.07)	0.142	0.83 (0.64, 1.08)	0.156
4–5 cups/day	0.78 (0.58, 1.04)	0.084	0.79 (0.58, 1.07)	0.128	0.81 (0.59, 1.10)	0.177
≥ 6 cups/day	0.59 (0.39, 0.89)	0.015	0.60 (0.38, 0.91)	0.020	0.59 (0.37, 0.91)	0.021
*P* for trend	0.015		0.024		0.031	

OR, odds ratio; CI, confidence interval. Model 1, adjusted for age, gender, BMI. Model 2: adjusted for model 1 covariates plus urban residence, geographical region, educational level, energy intake, smoking status, alcohol consumption, and other beverages consumption.

**Figure 3:**
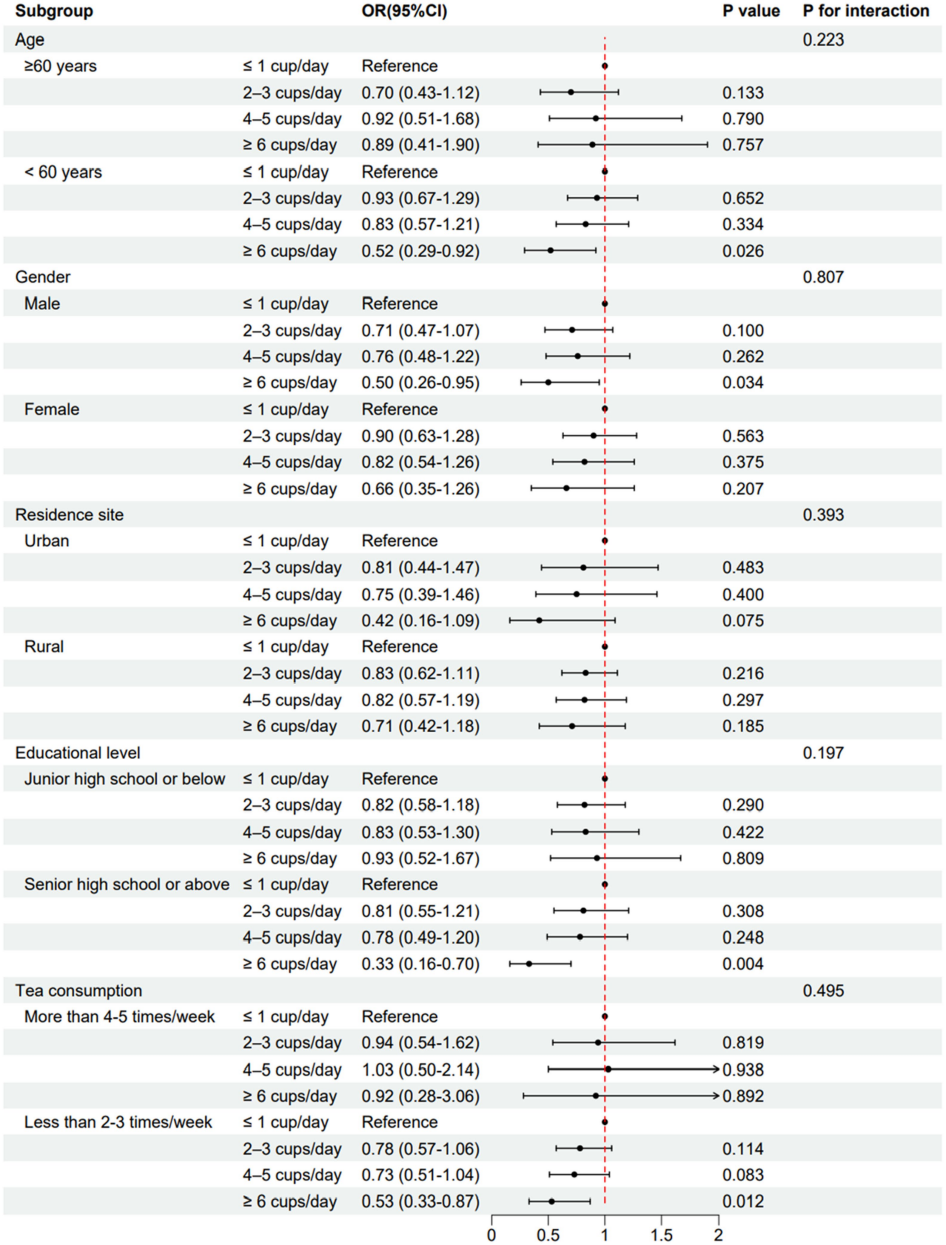
The association between plain water intake and hypertension in subgroups. Multivariate logistic analyses were performed in subgroups based on age (≥ 60 or < 60 years), gender (male or female), residence site (urban or rural), educational levels (junior high school or below, or senior high school or above) and tea consumption (more than 4–5 times/week or less than 2–3 times/week) after adjustments for covariates.

The results of stratified analyses were based on potential confounding factors including age, gender, residence site, educational level and tea intake (Figure 3). By stratifying age, there was only statistically significance of reduced risk of hypertension in ≥ 6 cups/day group in participants aged less than 60 years. Similarly, an inverse association with the risk of hypertension was found in participants who were male, who attained higher education and who consumed tea for less than 2–3 times/week. For participants living in urban residence, there was a similar trend in ≥6 cups/day group but without significance. In subgroup analyses, no interaction effect was found among these variables which indicated the results were relatively robust.

## 4 Discussion

To the best of our knowledge, this study was the first to investigate the association between plain water intake and hypertension in a large-scale, nationwide sample cohort among Chinese adults. In the present study, we found an inverse trend between plain water intake and the risk of hypertension over a follow-up of 9 years. Multivariate logistic regression analyses suggested that participants consuming ≥6 cups/day plain water had significantly lower risk of hypertension than those consuming ≤1 cup/day. In addition, subgroup analyses also found similar relationship in participants who aged less than 60 years, who were male, who attained higher education and who consumed tea for less than 2–3 times/week.

In a recent study published in *British Medical Journal*, Ma et al. found that replacing sugar-sweetened beverages with plain water could lower all cause and cardiovascular mortality in adults with type 2 diabetes (13). Their results highlighted the potential role of plain water to prevent cardiovascular disease risk in diabetic patients. Accumulating evidence has demonstrated that adequate water intake may be favorable for glycemic homeostasis and other health outcomes (16–19). Based on the evidence, the present study investigated the prospective association between plain water intake and the risk of developing hypertension. Our findings supported an independent role of drinking plain water ≥6 cups/day on reducing risk of hypertension. However, the attenuating effect of water intake on hypertension risk was only observed in participants consuming about 6–8 cups per day in restricted cubic spline model. Of note, too few participants consumed more than 8 cups per day, which may undermine statistical power to attain significance. In subgroup analyses, similar results were observed in participants aged less than 60 years, but not in older adults. It implied that low water intake may predispose youngers to develop hypertension later compare to the older adult. Gender differences also exist in this setting where males consuming ≥6 cups/day plain water had lower risk of hypertension compared with those consuming ≤1 cup/day. Interestingly, education attainment had a modifiable effect on the association between plain water intake and hypertension. It can be explained that individuals with the increase of education levels pay more attention in healthy lifestyles and health care. Plain water and tea intake account for the most part of daily fluid intake in Chinese adults. To minimize the influence of tea intake, we divided all participants into high tea consumers (more than 4–5 times/week) and low tea consumers (less than 2–3 times/week), and the results were generally consistent with the main findings in low tea consumers. Targeted water intake interventions in these subgroups might be more effective for the prevention of hypertension.

Plain water intake can be influenced by multiple factors, including age, gender, comorbidities, physical activity and environment (20, 21). In the present study, water intake was strongly associated with age, gender, residence and educational level at baseline. There was an increasing trend in the proportion of males and urban residence as plain water intake increased. Participants consuming more plain water tended to be younger and educated. In line with reason, participants consuming more plain water were likely to drink less tea. Generally, high plain water consumers are known to differ from low plain water consumers in many ways and more inclined to engage in other health-conscious behaviors, which may skew the observed association between plain water intake and hypertension. The beneficial effect of drinking adequate plain water requires confirmation from rigorously designed interventional studies.

Currently, the mechanisms underlying the relationship between plain water intake and hypertension are still elusive. In experimental studies, two disease animal models under conditions of pathological loss of free water developed arterial hypertension, which may be partly attributed to cutaneous vasoconstriction for limiting epidermal water loss (22, 23). Likewise, low water intake induces other type of dehydration, and leads to relative hyperosmolar milieu interne (24). The raised plasma osmolarity could increase blood pressure, both acutely and chronically (25). This state may involve activation of arginine vasopressin pathway which plays a crucial role in water reservation and vasoconstriction (26). Furthermore, maintaining appropriate hydration status is associated with metabolic improvement, which may modulate blood pressure to some extent (27–29). Given that, drinking adequate water seems to be protective from hypertension.

There were several limitations in the study. First, it was an observational study among Chinese adult residents, which was unable to confirm causal relationship between plain water intake and hypertension, and the results may not be generalized to other populations. Second, it was unavailable to eliminate the effects of potential confounding factors, such as family-related factors and socioeconomic status due to the limited information in this retrospective study. Third, there was nearly a decade elapsed since the cut-off date of our study design, which might result in a potential bias due to this time gap. Long-term studies of healthy water intake interventions are needed to evaluate the potential effect of optimal hydration status on hypertension in the future.

## 5 Conclusion

In summary, our findings suggested that there might be a favorable effect of plain water intake on preventing hypertension in a large cohort of Chinese adults from the general population. Drinking adequate amounts of plain water (about 6–8 cups/day) may reduce the risk of hypertension, particularly in the selected population. Further interventional studies are required to investigate the potential effect of increasing plain water intake on blood pressure regulation.

## Data availability statement

The original contributions presented in the study are included in the article/supplementary material, further inquiries can be directed to the corresponding author.

## Ethics statement

The studies involving humans were approved by the Institutional Review Board of the University of North Carolina at Chapel Hill, the National Institute for Nutrition and Food safety at China Center for Disease Control and Prevention, and the Human and Clinical Research Ethics Committee of the China-Japan Friendship Hospital. The studies were conducted in accordance with the local legislation and institutional requirements. Written informed consent for participation was not required from the participants or the participants’ legal guardians/next of kin in accordance with the national legislation and institutional requirements.

## Author contributions

SL: Data curation, Formal analysis, Writing – original draft. XX: Visualization, Writing – original draft. XZ: Conceptualization, Funding acquisition, Writing – review & editing.

## Footnotes

^1^ http://www.cpc.unc.edu/projects/china/home.html
